# Comparison of Clinical Features of COVID-19 vs Seasonal Influenza A and B in US Children

**DOI:** 10.1001/jamanetworkopen.2020.20495

**Published:** 2020-09-08

**Authors:** Xiaoyan Song, Meghan Delaney, Rahul K. Shah, Joseph M. Campos, David L. Wessel, Roberta L. DeBiasi

**Affiliations:** 1 Office of Infection Control and Epidemiology, Children’s National Hospital, Washington, DC; 2 Department of Pediatrics, George Washington University School of Health Science, Washington, DC; 3 Department of Laboratory Medicine, Children’s National Hospital, Washington, DC; 4 Division of Quality and Safety, Children’s National Hospital, Washington, DC; 5 Chief Medical Office, Children’s National Hospital, Washington, DC; 6 Division of Infectious Disease, Children’s National Hospital, Washington, DC

## Abstract

**Importance:**

Compared with seasonal influenza, the clinical features and epidemiologic characteristics of severe acute respiratory syndrome coronavirus 2 (SARS-CoV-2) and coronavirus 2019 (COVID-19) in US children remain largely unknown.

**Objective:**

To describe the similarities and differences in clinical features between COVID-19 and seasonal influenza in US children.

**Design, Setting, and Participants:**

This retrospective cohort study included children who were diagnosed with laboratory-confirmed COVID-19 between March 25 and May 15, 2020, and children diagnosed with seasonal influenza between October 1, 2019, and June 6, 2020, at Children’s National Hospital in the District of Columbia.

**Exposures:**

COVID-19 or influenza A or B.

**Main Outcomes and Measures:**

Rates of hospitalization, admission to the intensive care unit, and mechanical ventilator use and the association between underlying medical conditions, clinical symptoms, and COVID-19 vs seasonal influenza.

**Results:**

The study included 315 patients diagnosed with COVID-19 (164 [52%] male; median age, 8.3 years [range, 0.03-35.6 years]) and 1402 patients diagnosed with seasonal influenza (743 [53%] male; median age, 3.9 years [range, 0.04-40.4 years]). Patients with COVID-19 and those with seasonal influenza had a similar hospitalization rate (54 [17%] vs 291 [21%], *P* = .15), intensive care unit admission rate (18 [6%] vs 98 [7%], *P* = .42), and use of mechanical ventilators (10 [3%] vs 27 [2%], *P* = .17). More patients hospitalized with COVID-19 than with seasonal influenza reported fever (41 [76%] vs 159 [55%], *P* = .005), diarrhea or vomiting (14 [26%] vs 36 [12%], *P* = .01), headache (6 [11%] vs 9 [3%], *P* = .01), body ache or myalgia (12 [22%] vs 20 [7%], *P* = .001), and chest pain (6 [11%] vs 9 [3%], *P* = .01). Differences between patients hospitalized with COVID-19 vs influenza who reported cough (24 [48%] vs 90 [31%], *P* = .05) and shortness of breath (16 [30%] vs 59 [20%], *P* = .13) were not statistically significant.

**Conclusions and Relevance:**

In this cohort study of US children with COVID-19 or seasonal influenza, there was no difference in hospitalization rates, intensive care unit admission rates, and mechanical ventilator use between the 2 groups. More patients hospitalized with COVID-19 than with seasonal influenza reported clinical symptoms at the time of diagnosis.

## Key Points

**Question:**

What are the similarities and differences in clinical features between coronavirus disease 2019 (COVID-19) and seasonal influenza in US children?

**Findings:**

In this cohort study of 315 children with COVID-19 and 1402 children with seasonal influenza, there were no statistically significant differences in the rates of hospitalization, admission to the intensive care unit, and mechanical ventilator use between the 2 groups. More patients with COVID-19 than with seasonal influenza reported fever, diarrhea or vomiting, headache, body ache, or chest pain at the time of diagnosis.

**Meaning:**

The findings suggest that prevention of both COVID-19 and seasonal influenza in US children is prudent and urgent for the well-being of this population.

## Teaser

This cohort study compares the clinical features of coronavirus disease 2019 (COVID-19) with those of influenza A and B in US children.

## Introduction

For many years, public health officials have anticipated the emergence of a highly contagious respiratory virus with pandemic potential. When severe acute respiratory syndrome coronavirus 2 (SARS-CoV-2) began circulating in late 2019, it was immediately compared with both seasonal and pandemic influenza virus given the common features shared by these viruses. Both SARS-COV-2 and influenza viruses have demonstrated their ease of person-to-person transmission through the respiratory droplet route. The diseases caused by these viruses also share similar clinical presentations, including fever and respiratory symptoms that range from mild forms, such as cough, to severe lung infections.^1,2^ However, coronavirus disease 2019 (COVID-19) has also demonstrated distinct clinical characteristics, such as anosmia and hypogeusia.^3,4^ Although the search for a vaccine and a treatment continues for COVID-19, influenza has become detectable, vaccine preventable, and treatable.

Influenza in children has been well described and has been associated with serious complications including death.^5,6^ In contrast, there has been a severe paucity of pediatric data with regard to COVID-19. Early and limited data suggested that children may be less likely to contract COVID-19, with fewer of them requiring hospitalization or experiencing fever, cough, or shortness of breath, compared with adult patients.^7,8^ COVID-19–related pediatric death remains rare.^9^ However, new and severe clinical manifestations associated with COVID-19 continue emerging, including a rare but severe illness termed *multisystem inflammatory syndrome* in children.^10^

To date, little has been published directly comparing pediatric cohorts of patients with COVID-19 to those with seasonal influenza. In recognizing that prevention of COVID-19 may be further complicated during influenza season, when influenza and other respiratory viruses cocirculate in communities, we conducted this retrospective cohort study and compared children who were diagnosed with COVID-19, seasonal influenza A, and influenza B in a free-standing children’s hospital in Washington, DC.

## Methods

### Patient Population and Study Setting

This cohort study included children who were diagnosed with laboratory-confirmed COVID-19 between March 25 and May 15, 2020, and children who were diagnosed with laboratory-confirmed seasonal influenza between October 1, 2019, and June 6, 2020, at Children’s National Hospital in Washington, DC. Asymptomatic patients who tested positive for COVID-19 during preadmission or preprocedural screening were excluded from the study. The study was approved by the Children’s National Hospital institutional research board. Owing to the retrospective nature of this study, a waiver of consent was granted by the institutional review board. This study followed the Strengthening the Reporting of Observational Studies in Epidemiology (STROBE) reporting guideline.

Children’s National Hospital is a free-standing children’s hospital serving patients from communities in the District of Columbia, State of Maryland, and Commonwealth of Virginia, as well as other outlying areas. It has 2 large emergency departments, 12 community-based ambulatory clinics, and a hospital with 323 inpatient beds to provide acute and critical care to general pediatric and subspecialty care pediatric patients.

Since the patient with the first detected COVID-19 case presented for care to the Children’s National Hospital emergency department on March 15, 2020, patients have been continuously diagnosed with COVID-19 and treated in inpatient and outpatient settings, as described by a recent study.^11^

### Data Sources

Patients with COVID-19 were identified from a database maintained by the division of infectious disease for clinical management. Patients with influenza were identified from a laboratory informatics system. Hospitalized patients with influenza were obtained from a database maintained by the Office of Infection Control and Epidemiology. During influenza season, infection preventionists review medical charts to extract information as requested by the local department of health for the surveillance and reporting of patients hospitalized with influenza. Medical record review was performed to collect patients’ underlying medical conditions and symptoms at the time of diagnosis.

### Laboratory Testing

Testing for influenza A and B viruses was performed with 2 US Food and Drug Administration–cleared polymerase chain reaction assays: the Xpert Xpress Flu/RSV assay (Cepheid) and the ePlex Respiratory Pathogen Panel (GenMark Diagnostics). Testing for the SARS CoV2 was conducted using 1 of 3 molecular diagnostic assays that were approved under the Emergency Use Authorization by the US Food and Drug Administration. The 3 tests are used interchangeably as supplies are available: ePlex SARS-CoV-2 Test (GenMark Diagnostics), Simplexa COVID-19 assay (DiaSorin Molecular LLC), and Xpert Xpress SARS-CoV-2 (Cepheid).

### Statistical Analysis

We performed descriptive analysis and calculated a percentage for categorical variables and a mean or median for continuous variables. We used an unadjusted logistic regression method and calculated odds ratios (ORs) to assess statistical associations between independent variables and COVID-19 vs seasonal influenza. Subanalyses were conducted by comparing patients with COVID-19 with those with influenza types A and B. A 2-sided *P* < .05 was considered statistically significant. The analysis was conducted using Stata (StataCorp LLC).

## Results

Between March 25 and May 15, 2020, 315 patients (164 [52%] male; median age, 8.4 years [range, 0.03-35.6]) tested positive for COVID-19 at Children’s National Hospital. Of these patients, 54 (17%) required hospitalization, including 18 (6%) who required admission to the intensive care unit (ICU) and 10 (3%) who subsequently underwent mechanical ventilator treatment (Table 1).

**Table 1.** Comparison of Outcomes Among Patients With COVID-19, Influenza A, and Influenza B

Outcome	COVID-19	Seasonal influenza		
		A and B	A	B
Patients tested positive, No.	315	1402	674	728
Patients hospitalized, No. (%)	54 (17.1)	291 (20.8)	143 (21.2)	148 (20.3)
Patients requiring ICU stay, No. (%)	18 (5.7)	98 (7.0)	59 (8.8)	39 (5.4)
Patients requiring mechanical ventilator support, No. (%)	10 (3.1)	27 (1.9)	16 (2.4)	11 (1.5)
Hospital length of stay, mean (range), d	8.4 (1-45)	5.7 (1-100)	6.3 (1-100)	5.1 (1-58)
Mechanical ventilator support, median (range), d	10.1 (2-41)	7.0 (1-38)	8.1 (1-38)	5.4 (1-16)
Deaths, No. (%)	0	2 (0.1)	2 (0.3)	0

Abbreviation: COVID-19, coronavirus disease 2019; ICU, intensive care unit.

Between October 1, 2019, and June 6, 2020, 1402 patients (743 [53%] male; median age, 3.9 years [range, 0.03-40.4 years]) tested positive for influenza A or B. Of these patients, 291 (21%) were hospitalized, including 143 (49%) for influenza A and 148 (51%) for influenza B. Ninety-eight patients (7%) were admitted to the ICU, and 27 (2%) required mechanical ventilator support.

Compared with patients with seasonal influenza, patients with COVID-19 had a similar rate of hospitalization (54 [17%] vs 291 [21%]; OR, 0.8; 95% CI, 0.6-1.1; *P* = .15) and admission to the ICU (18 [6%] vs 98 [7%]; OR, 0.8; 95% CI, 0.5-1.3; *P* = .42); rates of mechanical ventilator support were also similar (10 [3%] vs 27 [2%]; OR, 1.5; 95% CI, 0.9-2.6; *P* = .17). Differences in duration of ventilator support between patients with COVID-19 vs those with seasonal influenza were not statistically significant (mean [SD], 10.1 [12.4] days vs 7.0 [7.9] days; *P* = .06).

No patients in this cohort were hospitalized with coinfection of both COVID-19 and seasonal influenza. During this study period, testing for influenza remained available for patients when clinically indicated, but a sharp decrease of influenza was detected at our facility after local school closures took place on March 15, 2020, followed by the activation of stay-at-home orders by local authorities on April 1, 2020.^12,13^ The positive detection rate for influenza decreased from 22% in the week ending on March 21, 2020, to 0.3% between March 22 and June 6, 2020, with only 1 influenza case detected.

 Two patients with influenza A died. No deaths were observed among patients with COVID-19 or influenza B.

### Comparison of Patients Hospitalized With COVID-19 With Those With Influenza A and B Combined

Patients hospitalized with COVID-19 (median age, 9.7 years [range, 0.06-23.2]) were older than those hospitalized with seasonal influenza (median age, 4.2 years [range, 0.04-23.1]). Patients older than 15 years accounted for 37% of those with COVID-19, in contrast to 6% in those with influenza (OR, 25.8; 95% CI, 14.2-48.5; *P* < .001) (Table 2).

**Table 2.** Comparison of Epidemiologic and Clinical Features of Patients Hospitalized With COVID-19, Influenza A, and Influenza B

Characteristic	COVID-19, No. (%) (n = 54)	Seasonal influenza								
		A and B			A			B		
		No. (%) (n = 291)	OR (95% CI)	*P* value	No. (%) (n = 143)	OR (95% CI)	*P* value	No. (%) (n = 148)	OR (95% CI)	*P* value
ICU admissions	18 (33)	98 (34)	1.0 (0.5-1.8)	1.0	59 (41)	0.7 (0.4-1.4)	.10	36 (24)	1.4 (0.7-2.7)	.30
Male	25 (46)	150 (52)	1.2 (0.7-2.2)	.50	77 (54)	0.7 (0.4-1.4)	.35	73 (49)	0.9 (0.5-1.7)	.70
Age, median (range), y	9.7 (0.06-23.20)	4.2 (0.04-23.10)	1.1 (1.1-1.2)	<.001	3.2 (0.07-23.2)	1.1 (1.1-1.2)	<.001	4.9 (0.04-22.1)	1.1 (1.0-1.2)	<.001
Age distribution, y										
<1	13 (24)	58 (20)	1 [Reference]	NA	29 (20)	1 [Reference]	NA	29 (20)	1 [Reference]	NA
1-4	7 (13)	104 (36)	0.3 (0.1-0.8)	.02	58 (41)	0.3 (0.1-0.8)	.01	46 (32)	0.34 (0.1-0.95)	.04
5-9	7 (13)	68 (23)	0.5 (0.2-1.2)	.12	30 (21)	0.5 (0.2-1.5)	.22	38 (26)	0.41 (0.2-1.2)	.09
10-14	7 (13)	43 (15)	0.7 (0.3-2.0)	.53	19 (13)	0.8 (0.3-2.4)	.72	24 (16)	0.65 (0.2-1.9)	.43
15-20	15 (28)	14 (5)	4.8 (1.9-12.3)	.001	4 (3)	8.4 (2.3-30.2)	.001	10 (7)	3.35 (1.2-9.4)	.02
>20	5 (9)	4 (1)	5.6 (1.3-23.7)	.02	3 (2)	3.7 (0.8-17.9)	.10	1 (0.7)	11.2 (1.2-105.2)	.04
Underlying medical condition present	35 (65)	121 (42)	2.6 (1.4-4.7)	.002	50 (35)	3.4 (1.8-6.6)	<.001	71 (48)	2.0 (1.0-3.8)	.04
Reported underlying medical condition										
Asthma	6 (11)	37 (13)	0.9 (0.3-2.1)	.74	19 (13)	0.8 (0.3-2.2)	.68	18 (12)	0.9 (0.3-2.4)	.90
Neurologic	11 (20)	24 (8)	2.8 (1.3-6.2)	.002	10 (7)	3.8 (1.5-9.4)	.004	14 (9)	2.4 (1.0-5.8)	.04
Cardiac	4 (7)	11 (4)	2.0 (0.6-6.7)	.24	3 (2)	3.7 (0.8-17.3)	.09	8 (5)	1.4 (0.4-4.9)	.60
Hematologic	5 (9)	22 (8)	1.3 (0.5-3.5)	.67	9 (6)	1.5 (0.5-4.8)	.47	13 (9)	1.1 (0.4-3.1)	.92
Oncologic	3 (6)	17 (6)	0.8 (0.3-3.4)	.95	8 (6)	0.99 (0.3-3.9)	.99	9 (6)	0.9 (0.2-3.5)	.89
Other	16 (30)	54 (19)	1.9 (1.0-3.6)	.07	16 (11)	3.3 (1.5-7.3)	.002	38 (26)	1.2 (0.6-2.4)	.57
Symptoms present at the time of diagnosis										
Fever	41 (76)	159 (55)	2.6 (1.4-5.1)	.005	69 (48)	3.4 (1.7-6.8)	.001	90 (61)	2.0 (1.0-4.1)	.05
Cough	24 (48)	90 (31)	1.8 (1.0-3.3)	.05	40 (28)	2.1 (1.1-3.9)	.03	50 (34)	1.6 (0.8-3.0)	.17
Congestion or runny nose	9 (17)	52 (18)	0.9 (0.4-2.0)	.83	23 (16)	1.0 (0.5-2.4)	.92	29 (20)	0.8 (0.4-1.9)	.64
Sore throat	3 (6)	6 (2)	2.8 (0.7-11.5)	.16	2 (1)	4.1 (0.7-25.5)	.13	4 (3)	2.1 (0.5-9.8)	.34
Shortness of breath	16 (30)	59 (20)	1.7 (0.9-3.2)	.13	32 (23)	1.5 (0.7-2.9)	.29	27 (18)	1.9 (0.9-3.9)	.08
Acute respiratory distress	3 (6)	27 (9)	0.6 (0.2-2.0)	.38	18 (13)	0.4 (0.1-1.5)	.17	9 (6)	0.9 (0.2-3.5)	.89
Diarrhea or vomiting	14 (26)	36 (12)	2.5 (1.2-5.0)	.01	15 (10)	3.0 (1.3-6.7)	.01	21 (14)	2.1 (1.0-4.5)	.05
Headache	6 (11)	9 (3)	3.9 (1.3-11.5)	.01	2 (1)	8.8 (1.7-45.1)	.01	7 (5)	2.5 (0.8-7.9)	.11
Body ache or myalgia	12 (22)	20 (7)	3.9 (1.8-8.5)	.001	6 (4)	6.5 (2.3-18.4)	<.001	14 (9)	2.7 (1.2-6.4)	.02
Chest pain	6 (11)	9 (3)	3.9 (1.3-11.5)	.01	2 (1)	8.8 (1.7-45.1)	.01	7 (5)	2.5 (0.8-7.9)	.11

Abbreviations: COVID-19, coronavirus disease 2019; ICU, intensive care unit; NA, not applicable; OR, odds ratio.

Of patients hospitalized with COVID-19, 35 [65%] had at least 1 underlying medical condition, significantly higher than the 121 [42%] observed in those hospitalized with influenza (OR, 2.6; 95% CI, 1.4-4.7; *P* = .002). Neurological issues owing to global developmental delay or seizures were the most often identified underlying condition and were present in 11 patients (20%) hospitalized with COVID-19 compared with 24 patients (8%) hospitalized with influenza (OR, 2.8; 95% CI, 1.3-6.2; *P* = .002). There was no statistically significant difference in patients reporting a history of asthma, cardiac, hematologic, and oncologic conditions in those with COVID-19 vs those with influenza.

In both groups, fever was the most often reported symptom at the time of diagnosis followed by cough. A greater proportion of patients hospitalized with COVID-19 than those hospitalized with seasonal influenza reported fever (41 [76%] vs 159 [55%]; OR, 2.6; 95% CI, 1.4-5.1; *P* = .01), diarrhea or vomiting (14 [26%] vs 36 [12%]; OR, 2.5; 95% CI, 1.2-5.0; *P* = .01), headache (6 [11%] vs 9 [3%]; OR, 3.9; 95% CI, 1.3-11.5; *P* = .01), body ache or myalgia (12 [22%] vs [7%]; OR, 3.9; 95% CI, 1.8-8.5; *P* = .001), and chest pain (6 [11%] vs 9 [3%]; OR, 3.9; 95% CI, 1.3-11.5; *P* = .01). Differences in those reporting cough were not statistically significant (24 [48%] vs 90 [31%]; OR, 1.8; 95% CI, 1.0-3.3; *P* = .05).

No statistically significant difference was found in the number of patients hospitalized with COVID-19 vs those with seasonal influenza who reported congestion (9 [17%] vs 52 [18%]; OR, 0.9; 95% CI, 0.4-2.0; *P* = .93), sore throat (3 [6%] vs. 6 [2%]; OR, 2.8; 95% CI, 0.7-11.5; *P* = .16), and shortness of breath (16 [30%] vs 59 [20%]; OR, 1.7; 95% CI, 0.9-3.2; *P* = .13).

### Comparison of Patients Hospitalized With COVID-19 With Those With Influenza A and Influenza B

Compared with patients hospitalized with influenza A, more patients hospitalized with COVID-19 reported fever (41 [76%] vs 69 [48%]; OR, 3.4; 95% CI, 1.7-6.8; *P* = .001), cough (24 [48%] vs 40 [28%]; OR, 2.1; 95% CI, 1.1-3.9; *P* = .03), diarrhea and vomiting (14 [26%] vs 15 [10%]; OR, 3.0; 95% CI, 1.3-6.7; *P* = .01), and body ache or myalgia (12 [22%] vs 6 [4%]; OR, 6.5; 95% CI, 2.3-18.4; *P* < .001).

In contrast, when patients hospitalized with COVID-19 were compared with those with influenza B, no statistically significant differences were found in those reporting fever (41 [76%] vs 90 [61%]; OR, 2.0; 95% CI, 1.4-4.1; *P* = .05), cough (24 [48%] vs 50 [34%]; OR, 1.6; 95% CI, 0.8-3.0; *P* = .17), diarrhea or vomiting (14 [26%] vs 21 [14%]; OR, 2.1; 95% CI, 0.99-4.54; *P* = .05), and headache and chest pain (6 [11%] vs 7 [5%]; OR,. 3.9; 95% CI, 1.3-11.5; *P* = .11).

## Discussion

Much remains unknown about SARS-CoV-2, but it has infected approximately 7 million people worldwide,^14^ including at least 2 million US individuals.^15^ With a mean mortality rate of 7%,^14^ nearly 400 000 people have died of COVID-19 worldwide, with over 110 000 reported in the US alone by May 23, 2020.^15^ These reported deaths have exceeded the estimated annual deaths of seasonal influenza.^16^

Overall, in this cohort of pediatric patients with COVID-19 or seasonal influenza, we found no statistically significant differences in hospitalization rate, ICU admission rate, and use of mechanical ventilator support between the 2 groups.

Among those hospitalized with COVID-19, patients exhibited clinical symptoms that were similar to those reported in previous studies, including 76% reporting fever and 48% reporting cough.^17,18^ About one-quarter of patients hospitalized with COVID-19 reported shortness of breath, diarrhea or vomiting, and body ache or myalgia.

Overall, more patients hospitalized with COVID-19 than those with seasonal influenza reported clinical symptoms, including fever, diarrhea or vomiting, headache, body ache or myalgia, and chest pain, at the time of diagnosis.

Compared with patients hospitalized with seasonal influenza, we found more patients hospitalized with COVID-19 that were older than 15 years or had underlying medical conditions. Recognizing that the upcoming influenza season could occur with COVID-19 cocirculating in the community, we believe it is prudent to ensure individuals with comorbidities receive the influenza vaccine to prevent severe disease courses that lead to hospitalization.

During the study period, we observed an abrupt decrease in influenza cases detected in our facility after local governments announced school closures and stay-at-home orders to combat the community spread of COVID-19. The decrease in influenza cases might have contributed to the observation that none of the patients in the study cohort were hospitalized for coinfection of COVID-19 and seasonal influenza. Coinfections of COVID-19 with influenza and other respiratory viruses have been reported previously.^19,20^ Further study is warranted to assess the potential benefits of interventions such as school closure on reducing disease burden and maintaining optimal operation of health care facilities.

### Limitations

This study has limitations. Because it was a retrospective study, the findings are subject to biases owing to recall error or missing information that were introduced during a patient encounter. A single-center study also limited our ability to conduct subcohort analysis on patients who were admitted to the ICU because of an insufficient sample size and limited our ability to assess other risk factors, such as obesity. This study was also limited by differences in case ascertainment through positive test results because the 2 viruses were detected by different platforms.

## Conclusions

This study found that patients with COVID-19 and those with seasonal influenza had similar hospitalization rates, ICU admission rates, and mechanical ventilator use. Compared with patients hospitalized with seasonal influenza, a greater proportion of patients hospitalized with COVID-19 had underlying medical conditions and reported fever, diarrhea or vomiting, headache, body ache or myalgia, or chest pain. As the COVID-19 pandemic continues and the 2020-2021 influenza season approaches, findings from our study may inform the prompt identification and treatment of children with a respiratory viral infection in health care facilities.
